# Selective Small Molecule Compounds Increase BMP-2 Responsiveness by Inhibiting Smurf1-mediated Smad1/5 Degradation

**DOI:** 10.1038/srep04965

**Published:** 2014-05-14

**Authors:** Yu Cao, Cheng Wang, Xueli Zhang, Guichun Xing, Kefeng Lu, Yongqing Gu, Fuchu He, Lingqiang Zhang

**Affiliations:** 1 State Key Laboratory of Proteomics, Beijing Proteome Research Center, Beijing Institute of Radiation Medicine, Collaborative Innovation Center for Cancer Medicine, Beijing, China; 2 School of Medicine, Shihezi University, Shihezi, Xinjiang Province, China; 3 Institute of Cancer Stem Cell, Dalian Medical University, Dalian, Liaoning Province, China

## Abstract

The ubiquitin ligase Smad ubiquitination regulatory factor-1 (Smurf1) negatively regulates bone morphogenetic protein (BMP) pathway by ubiquitinating certain signal components for degradation. Thus, it can be an eligible pharmacological target for increasing BMP signal responsiveness. We established a strategy to discover small molecule compounds that block the WW1 domain of Smurf1 from interacting with Smad1/5 by structure based virtual screening, molecular experimental examination and cytological efficacy evaluation. Our selected hits could reserve the protein level of Smad1/5 from degradation by interrupting Smurf1-Smad1/5 interaction and inhibiting Smurf1 mediated ubiquitination of Smad1/5. Further, these compounds increased BMP-2 signal responsiveness and the expression of certain downstream genes, enhanced the osteoblastic activity of myoblasts and osteoblasts. Our work indicates targeting Smurf1 for inhibition could be an accessible strategy to discover BMP-sensitizers that might be applied in future clinical treatments of bone disorders such as osteopenia.

BMPs promote differentiation of mesenchymal stem cells into osteoblasts and stimulate osteogenesis activity, thus can be utilized clinically to increase bone synthesis activity in osteopenia treatment and fracture repair^1^,^2^. In 2002, recombinant human BMP-2 (rhBMP-2) was approved by the Food and Drug Administration (FDA) as a bone graft substitute to achieve fracture repair and spinal fusion^3^. However, the effective concentration of rhBMP-2 for human use is over ten thousand times higher than that of cultured cells^4^,^5^,^6^, which makes rhBMP-2 hardly replaceable to current drugs due to its high manufacturing costs. To overcome this difficulty, we developed a strategy aiming to accelerate BMP signaling efficiency and to increase consequent responsiveness under unit stimulation.

BMP signals are canonically transferred by the Signaling Mothers Against Decapentaplegic (Smad) cascade. Firstly, the receptor-activated Smads, Smad1 and Smad5 (or Smad1/5) are phosphorylated, and then form a transcriptional complex with Smad4. The complex is further translocated into the nucleus and regulates expression of BMP-target genes^7^,^8^. After the signal is transferred, components such as Smad1/5 are destined to destruction to prevent the mild BMP signal from bursting into an overwhelming consequence^9^. The HECT (Homologous to the E6-AP Carboxyl Terminus) type ubiquitin ligase Smurf1 was identified as a negative regulator of BMP signaling, as it ubiquitinates Smad1 and Smad5 for proteasomal degradation^10^. Smurf1 knockout mice exhibited increased bone mineral density (BMD) without other distinct phenotypes^11^. In accord with this, the Smurf1 transgenic mice showed significant bone formation attenuation during postnatal life^12^. The negative role of Smurf1 in BMP signaling, along with the evidence of age-dependent bone mass increase of Smurf1 null mice indicates that Smurf1 is an eligible target for inhibition to increase BMP signaling activity.

Smad1/5 undergoes a second series of phosphorylation by cyclin-dependent kinase-8 (CDK8) and glycogen synthase kinase-3 (GSK3) after their binding to the genome. CDK8-mediated phosphorylation of Smad1/5 facilitates the transcriptional complex in activating its target genes. Furthermore, it promotes GSK3-mediated phosphorylation of Smad1/5, which leads to the capture of Smad1/5 by Smurf1^13^. Smad1/5 contains a PY motif (D221-P229) and four CDK8/GSK3 phosphorylation sites (S206 and S214 for CDK8; T202 and S210 for GSK3) in the linker region between MH1 and MH2 domains. When capturing Smad1/5, Smurf1 WW2 domain interacts with the PY motif while the WW1 domain binds to the linker region with phosphorylation sites (S210 and S214). Previous study revealed that diphosphorylated Smad1 on S210 and S214 sites has much higher binding affinity with Smurf1, compared with S210 mono-phosphorylated Smad1^13^. The higher affinity of WW1 domain to the diphosphorylated Smad1 indicates that the WW1 domain of Smurf1 is key to recognize specific substrates which need to be degraded^13^.

In this study, we defined a potential ligandable pocket on Smurf1 WW1 domain as a docking site for small molecule compounds and performed virtual docking (see Methods). We then developed a series of tests to specifically select bio-active compounds, and identified two compounds which stabilize Smad1/5 protein level via inhibiting Smurf1-mediated ubiquitination of Smad1/5 when the cells were stimulated by rhBMP-2. Supportively, expression of BMP signaling downstream genes and ALP activity in both mouse myoblasts and osteoblasts were increased after administrated those two compounds with a low dose. These results suggest that targeting Smurf1 for inhibition is an accessible method to increase BMP signaling responsiveness. Moreover, our study established a bio-active compounds selection approach including both *in silico* virtual screening and experimental assays, which is a proof of principle to discover small molecule compounds that antagonize HECT type ubiquitin ligase and design drugs specifically to increase the cellular signal transduction efficiency.

## Results

### *In silico* screening for Smurf1-targeting compounds

**Figure 1:**
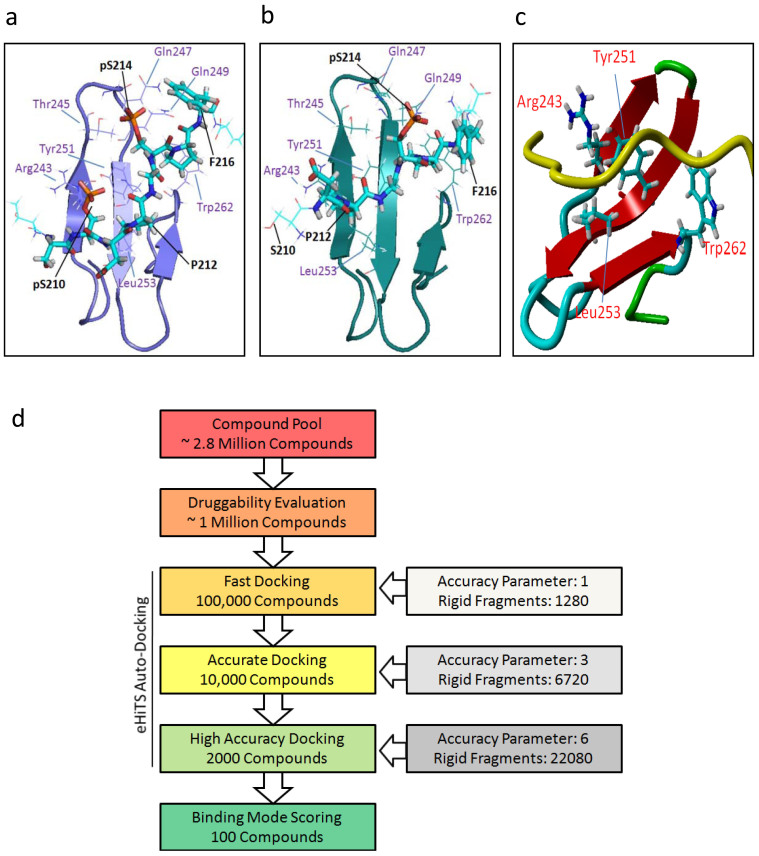
Binding pocket definition and *in silico* screening. (a) Refined structure of the Smurf1 WW1 domain bound to the diphosphorylated (pS210/pS214) region of the Smad1 linker. Key residues in Smad1 (black) and Smurf1 (purple) are labeled. (b) Refined structure of the Smurf1 WW1 domain bound to the monophosphorylated (pS214) region of the Smad1 linker. Key residues in Smad1 (black) and Smurf1 (purple) are labeled. (c) Receptor model of the Smurf1 WW1 domain (multiple colored) and diphosphorylated (pS210/pS214) region of the Smad1 linker (yellow). Key residues important for interactions are highlighted, and the region encompassed in those residues was defined as the binding pocket. (d) Workflow of *in silico* screening.

To bind to the diphosphorylated region of Smad1, Tyr251 and Arg243 on Smurf1 WW1 domain coordinate the phosphorylated group of pS210 via a hydroxyl and a guanidino group, respectively; while side chains of Thr245, Gln247 and Gln249 contact pS214 on the phosphorylated group (Figure 1a). In contrast, when S210 is not phosphorylated, residues on the WW1 domain have less contact with this residue, which leads to a lower binding affinity of Smurf1 to Smad1. Although D211 coordinates the side chains of Arg243, this interaction is different from what pS210 conducts and subsequently leads to a weakened contact between pS214 site and the WW1 domain (Figure 1b). Based on the structural information of Smurf1-Smad1 interactions, we defined an area on the WW1 domain, surrounded by R243, Y251, L253 and W262, as a hydrophobic pocket which might satisfy the requirement of accommodating small chemical molecules in the concave groove (Figure 1c). Next, we performed computer based high-throughput virtual screening by a rigorous workflow (Figure 1d, also see Methods). We screened more than one million diverse and commercially available small molecule compounds and obtained approximately 2000 compounds after the virtual docking. Next, we evaluated these hits from the in silico docking results after visualized different poses by CheVi (SimBioSys Inc. Canada) for two criteria: a). whether the hits directly interact with the defined pocket and key residues; b). whether the binding conformation is stretched.

### Primary selection of bio-active compounds

**Figure 2:**
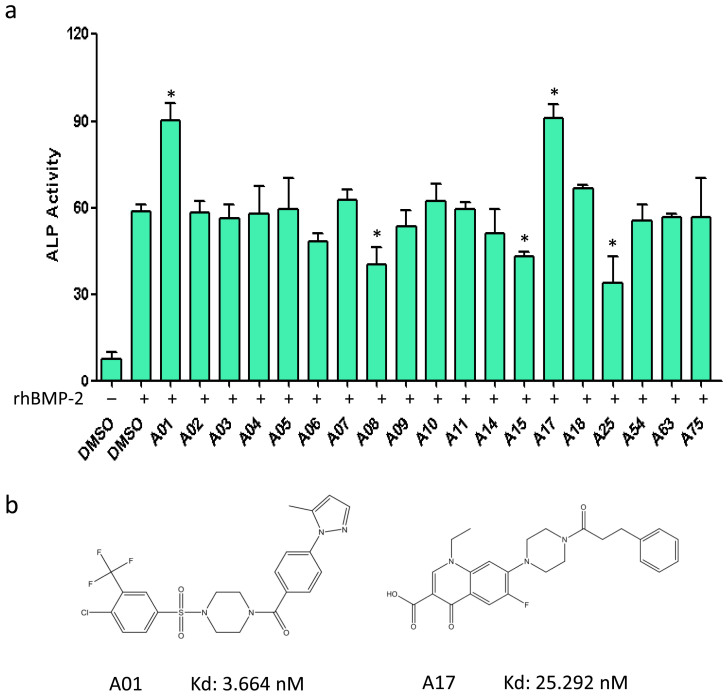
Primary selection of potential bio-active compounds. (a) Determination of the efficacy of various selected compounds of enhancing BMP induced ALP activity. Compounds were added at a concentration of 2 μM, while rhBMP-2 was used at 50 ng/ml. The compound concentration for screening was selected empirically. In DMSO controls, cells were treated with DMSO (0.01%) instead of compounds (same for the following figures). Data points were determined in triplicate and showed with the mean ± SD (*: p < 0.05, t-test). (b) Chemical structures of A01 and A17.

Through binding mode evaluation and compounds skeleton clustering, 19 hits out of the top 100 scoring ones were chosen for experimental selection (supplementary Table S1). ALP activity assay was employed as the unidimensional elimination standard, with the purpose of sifting out functional candidates for further evaluation. Mouse myoblasts cell line C2C12, derived from mouse mesenchymal stem cell, was cultured for cellular examinations due to its strong response to BMP signaling. After a low-dose treatment (1 μM), A01 and A17 were considered to be selective candidates for their positive effects on ALP activity under BMP-2 stimulation (Figure 2a). Other chemicals were excluded since their effects on ALP activity were not significant or negative. Then, *in vitro* Smurf1-Smad1 binding assay was performed to test whether selected compounds fit the hypothesis described previously. Ten among the “not negative” compounds from ALP assay were chose to get tested; and the result showed A01 and A17 could interrupt Smurf1-Smad1 interaction (Supplementary Figure S1). A01 and A17 have distinct chemical structures, which may lead to their different contacting manners with the target docking pocket. Both compounds' properties fit the rules of Lipinski and have low predicted dissociation constants (Kd) of chemical-protein interactions (Figure 2b and Supplementary Table S2).

### Selective compounds stabilize endogenous Smad1/5 protein level

**Figure 3:**
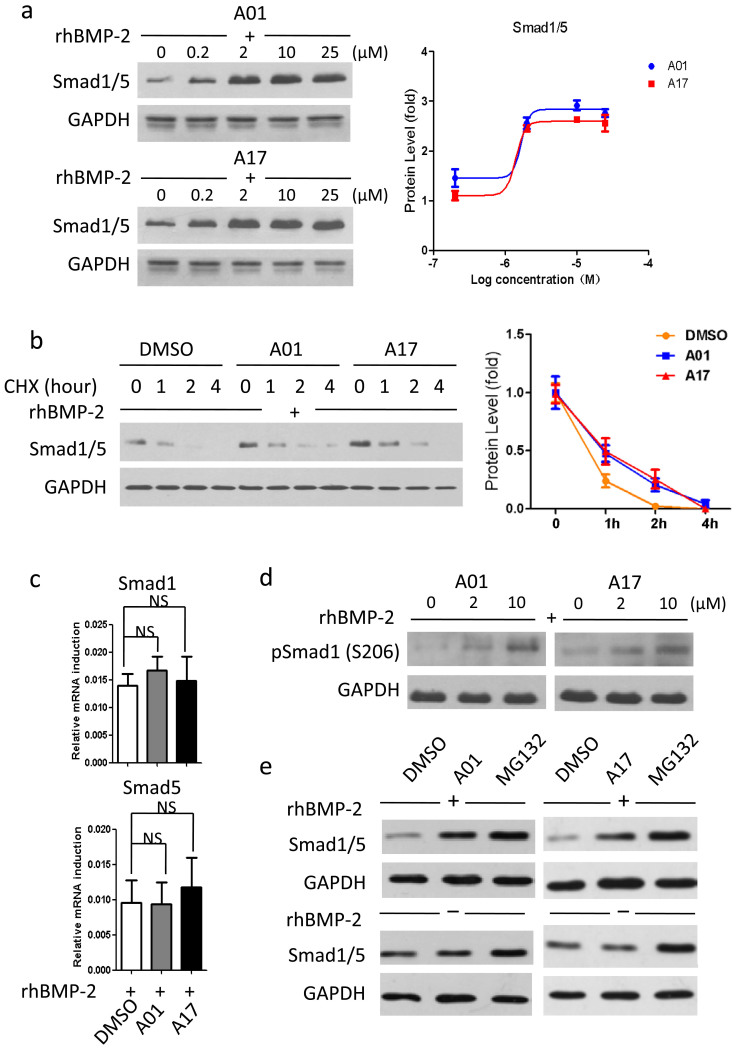
Selective compounds stabilize Smad1/5 protein level. (a) Selective compounds stabilized Smad1/5 protein level. C2C12 cells were treated A01 and A17 at incremental concentrations, while rhBMP-2 was used at 50 ng/ml. For the zero concentration, cells were treated with DMSO (0.01%) instead of compounds (same for the following figures). GAPDH were used as loading controls. Note that cropped blots are shown here. Left graph shows densitometry levels of percent Smad1/5 (normalized by GAPDH and compared with vehicle groups) and was fitted by GraphPad Prism 5. (b) Selective compounds prolonged Smad1/5 protein decay. C2C12 cells were treated with cycloheximide (CHX, 10 μg/ml), rhBMP-2 (50 ng/ml) and selected compounds (2 μM). Cells were collected at different time points. GAPDH were used as loading controls. Note that cropped blots are shown here. Left graph shows densitometry levels of percent Smad1/5 (normalized by GAPDH) and was fitted by GraphPad Prism 5. (c) Selective compounds had insignificant impact on Smad1 and Smad5 mRNA levels. C2C12 cells were treated A01 and A17 at 2 μM, while rhBMP-2 was used at 50 ng/ml. Data points were determined in triplicate and showed with the mean ± SD (NS: p > 0.05, t-test). (d) Selective compounds stabilized Smad1 phosphorylation (S206) level. C2C12 cells were treated A01 and A17 at 2 μM and 10 μM, while rhBMP-2 was used at 50 ng/ml. GAPDH were used as loading controls. Note that cropped blots are shown here. (e) Detection of Smad1/5 protein level following selective compounds or proteasome inhibitor treatments. C2C12 cells were treated with A01 and A17 at 2 μM or MG132 at 20 μM, while rhBMP-2 was absent or present (used at 50 ng/ml). GAPDH were used as loading controls. Note that cropped blots are shown here.

Further investigations focused on whether selective candidates function via BMP-Smad1/5 cascade and affect Smurf1-mediated Smad1/5 degradation. Under rhBMP-2 stimulation, C2C12 cells were administrated with A01 and A17 at graded concentrations simultaneously, and the subsequent test revealed that endogenous Smad1/5 protein level reserved in a dose-dependent manner compared with control group (Figure 3a). 8 hours was chosen as the administration span to examine the protein level because that after stimulated by rhBMP-2, Smad1/5 protein level declined time-dependently and bottomed after approximate eight hours (Supplementary Figure S2); this observation was different from a former report^14^. Then, protein decay assay was performed and the result showed that A01 and A17 could prolong the half-life of endogenous Smad1/5 about 2 hours under rhBMP-2 stimulation (Figure 3b). In addition, the mRNA levels of Smad1 and Smad5 were not significantly changed under the same treatment conditions, suggesting that A01 and A17 both affect Smad1/5 protein level (Figure 3c). Further, the level of S206 phosphorylated Smad1 was tested and confirmed the effect of A01 and A017 on reserving the phosphorylated Smad1 (Figure 3d). S206 phosphorylation of Smad1 is essential for its Smurf1-mediated degradation; thus, the reserved S206 phosphorylated Smad1 might be still active as the signal mediator. Then A01 and A17 administrations were compared with treatment of the proteasome inhibitor MG132, which inhibits protein ubiquitin-proteasome degradation indistinguishably. Under rhBMP-2 stimulation, treatment of A01 and A17 was able to reserve Smad1/5 protein level quite close to that of MG132 (Figure 3e). However, without rhBMP-2, the level of Smad1/5 was also increased visibly after treated with MG132, indicating that a Smurf1-independent mechanism of degrading non-activated Smad1/5 might be existent.

### Selective compounds impair Smurf1-mediated Smad1/5 degradation

**Figure 4:**
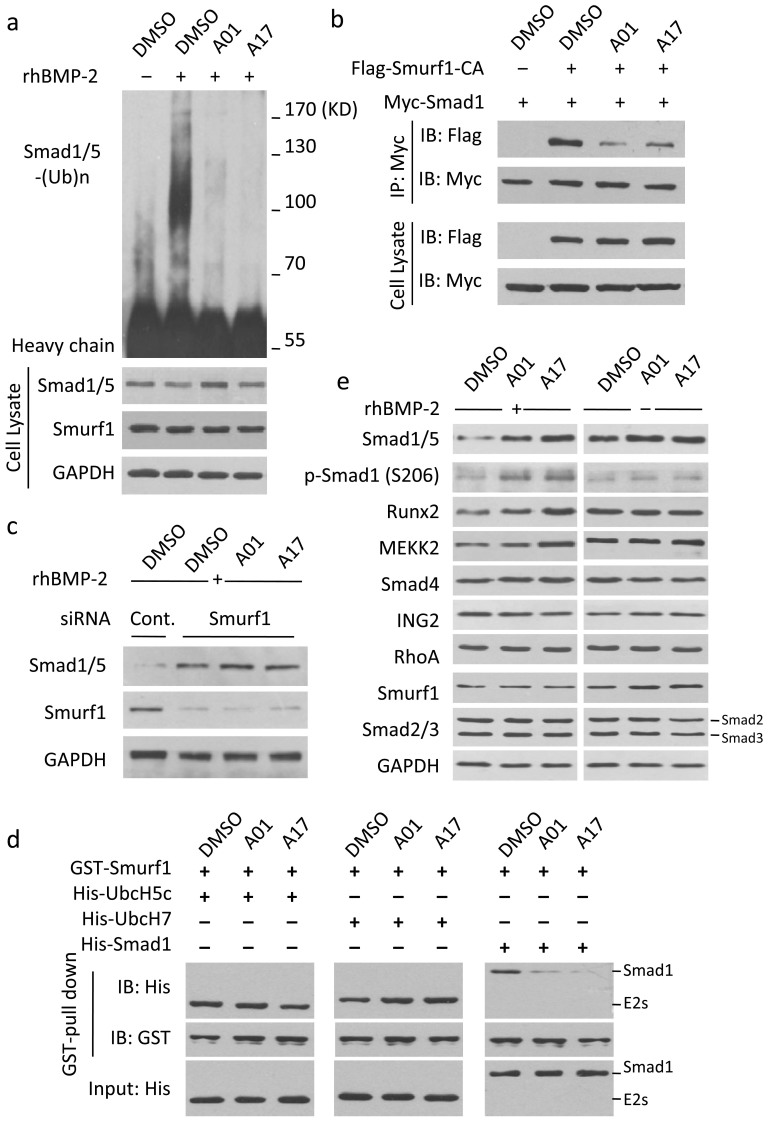
Selective compounds impair Smurf1-mediated Smad1/5 degradation. (a) Selective compounds impeded the ubiquitination of Smad1/5. C2C12 cells were treated A01 and A17 at 2 μM, while MG132 and rhBMP-2 were used at 20 μM and 50 ng/ml. GAPDH were used as loading controls. Note that cropped blots are shown here. (b) Selective compounds blocked Smurf1-Smad1 interaction. 293T cells were co-transfected Flag-empty vector and Myc-Smad1 (lane 1) or Flag-Smurf1-CA and Myc-Smad1 (lane 2–4) plasmids. For inhibitors administration, cells were treated A01 and A17 at 2 μM. Note that cropped blots are shown here. (c) Selective compounds had unnoted effects on Smad1/5 without Smurf1. Smurf1 was knocked down in C2C12 cells (lane1: control siRNA, lane2–4: mouse Smurf1 siRNA). For inhibitors administration, cells were treated A01 and A17 at 2 μM, while rhBMP-2 was used at 50 ng/ml. GAPDH were used as loading controls. Note that cropped blots are shown here. (d) Selective compounds impaired Smurf1-Smad1 but not Smurf1-E2s interaction. Prokaryotic expressed proteins were purified and employed in GST-pull down. A01 and A17 were used at 10 μM. Note that cropped blots are shown here. (e) Effects of selective compounds on Smurf1 substrates and TGF-β pathway components. C2C12 cells were treated A01 and A17 at 2 μM, while rhBMP-2 was used at 50 ng/ml. GAPDH were used as loading controls. Note that cropped blots are shown here.

To test whether the selective compounds A01 and A17 stabilized Smad1/5 protein level by preventing their ubiquitin-proteasome mediated degradation; an *in vivo* Smad1/5 ubiquitination assay was performed. A01 and A17 strongly inhibit Smad1/5 ubiquitination under rhBMP-2 stimulation comparing to the control (Figure 4a). Moreover, to further examine that the compounds function by impairing Smad interactions with their ubiquitin ligase Smurf1, *in vivo* assay of Smad1-Smurf1 interaction was performed. Exogenous Smad1 and Smurf1 were transfected into HEK293T cells; and co-immunoprecipitation of Smad1 showed that both selective compounds partially attenuated Smad-Smurf1 interaction (Figure 4b). Although Smurf1 tends to target activated Smad1/5 to terminate their function, Smurf1 over-expression could also lead Smurf1-Smad1/5 interaction and mediate smad1/5 degradation without BMP stimulation; it may be due to the excessive abundance of exogenous Smurf1 would ignore the inapposite conformation of the substrate and the lower binding affinity. Hence, the result showed in Figure 4b should be in line with former conclusions. Under rhBMP-2 stimulation, A01 and A17 administration had no effects on Smad1/5 protein level when Smurf1 was knocked down by specific siRNA (Figure 4c); this result indicates that those compounds might only affect Smad1/5 level in a Smurf1 dependent manner. In line with this, in vitro assays revealed that A01 and A17 could elevate Smad1/5 when cells were pre-transfected wild type Smurf1 but not the C699A (Smurf1 CA) mutant which lost the ubiquitin ligase activity (Supplementary Figure S3). Smuf1 has two E2s, UbcH5c and UbcH7, which interact with the HECT domain and deliver the ubiquitin onto it. To rule out the possibility that the selected compounds may function by blocking Smurf1-E2 interaction, *in vitro* binding assay was performed. Both compounds could interrupt Smurf1-Smad1 but not Smurf1-E2 contacts (Figure 4d and Supplementary Figure S4). Next, the impacts of selected compounds on some other Smurf1 substrates and components of TGF-β signal pathway with or without BMP-2 stimulation were examined (Figure 4e). Both A01 and A17 did not affect the protein level of Smad2/3, Smad4, ING2 and the small guanosine triphosphatase RhoA. Although ING2, RhoA and Smad4 were reported as substrates of Smurf1, the binding modes between them and Smurf1 are not canonical WW-PY interaction^15^,^16^,^17^,^18^,^19^; this result suggests that both compounds may specifically block WW domains mediated Smurf1-substrate interaction; However, both selected compounds only exerted mild positive effects on protein stability of Runx2 and MEKK2, which have been demonstrated to be captured by WW-PY interaction. Further investigation showed that both compounds slightly prolonged protein half-life under BMP-2 stimulation (Supplementary Figure S5). It may because the Smurf1-mediated degradation of Runx2 does not fully require BMP-2 stimulation^20^. Although the observed effects were mild, they might indicate the possibility that selective compounds act partly through blocking Smurf1 mediated Runx2 degradation. Ubiquitin ligase Smurf2, the homologous protein of Smurf1, captures Smad2/3 via the WW2 and WW3 domains and promotes the ubiquitin-proteasome degradation of Smad2/3; this process inhibits TGF-β signaling^21^,^22^. To examine the target specificity of the selective compounds, the effects of both compounds on the interactions of Smurf2-Smad2/3 were tested via *in vivo* co-immunoprecipitation. The results showed that A01 and A17 could not interrupt Smurf2-Smad2 or Smurf2-Smad3 interactions (Supplementary Figure S6), indicating that both compounds might act specifically on Smurf1.

### Selective compounds enhance BMP signaling responsiveness

**Figure 5:**
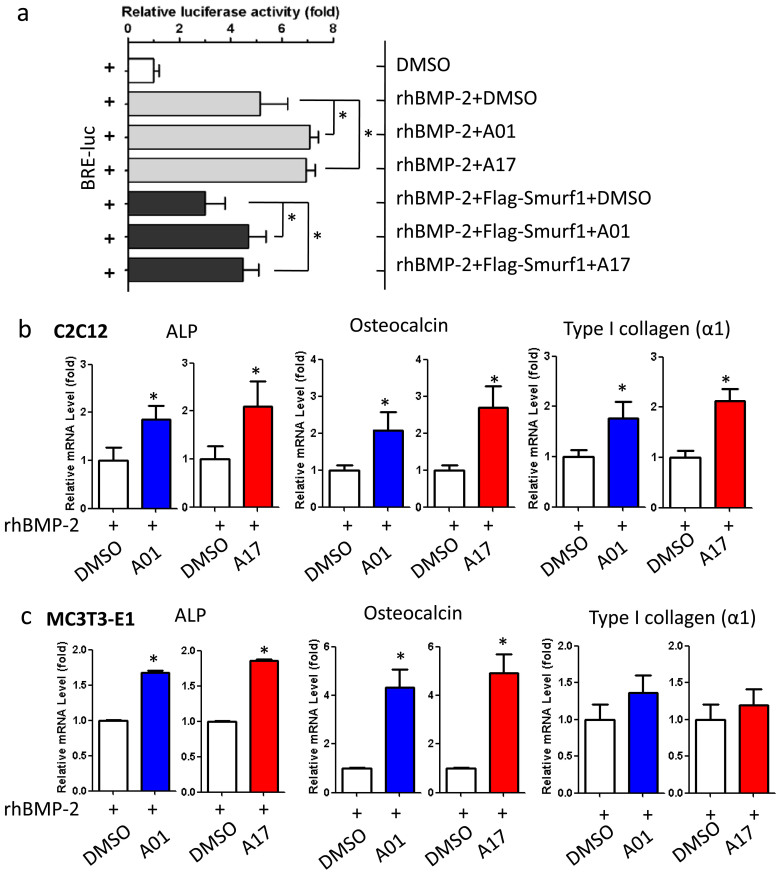
Selective compounds enhance BMP-2 signaling responsiveness. (a) Selective compounds increased BMP-2 responsible reporter gene activity. Data points were determined in triplicate and showed with the mean ± SD (*: p < 0.05, t-test). (b) Selective compounds increased ALP, osteocalcin and type I collagen (α1) expression in mouse myoblasts. C2C12 cells were treated A01 and A17 at 2 μM, while rhBMP-2 was used at 50 ng/ml. Data points were determined in triplicate and showed with the mean ± SD (*: p < 0.05, t-test). (c) Selective compounds increased ALP, osteocalcin and type I collagen (α1) expressions in mouse pre-osteoblasts. MC3T3-E1 cells were treated A01 and A17 at 2 μM, while rhBMP-2 was used at 50 ng/ml. Data points were determined in triplicate and showed with the mean ± SD (*: p < 0.05, t-test).

Next, the effects of selected compounds on BMP signaling responsiveness was firstly delineated by luciferase reporter assay (Figure 5a). BMP-2 treatment remarkably stimulated the BMP-responsive BRE-luc (BMP-2 responsible element) activity, and treatments of A01 and A17 could further increase this activity. Smurf1 over-expression inhibited BMP-2 mediated elevation of BRE-luc luciferase activity, but A01 and A17 could rescue the reduced responses. Although BMP signaling controls multiple biological and physiological processes, here, we focused on osteoblastic activity induced by BMP-2. The gene expression of ALP, osteocalcin and type I collagen (α1) was detected after administrating A01 and A17 to C2C12 and mouse pre-osteoblasts MC3T3-E1 cells. Comparing with the control group, both compounds increased the expression of BMP-2 downstream genes (Figure 5b–c). Elevated mRNA levels of ALP, osteocalcin and type I collagen (α1) indirectly revealed the increased responsiveness to BMP-2 stimulation. In C2C12 cells, selective compounds elevated the expression of those genes by about 1.5 to 2 folds; but in MC3T3-E1 cells, they elevated ALP mRNA level by more than 1.5 fold and osteocalcin by about 4 fold while had insignificant effects on type I collagen (α1) mRNA level. To examine the specific effects of A01 and A17 on BMP induced ALP, osteocalcin and collagen, the mRNA level of them were detected after cells were treated with compounds alone or with BMP-2 (Supplementary Figure S7). Without BMP-2 stimulation, all tested target genes expressed at low abundance, and both selected compounds exerted no effect on them upon administration.

### Selective compounds potentiate BMP-2 induced osteoblastic activity

**Figure 6:**
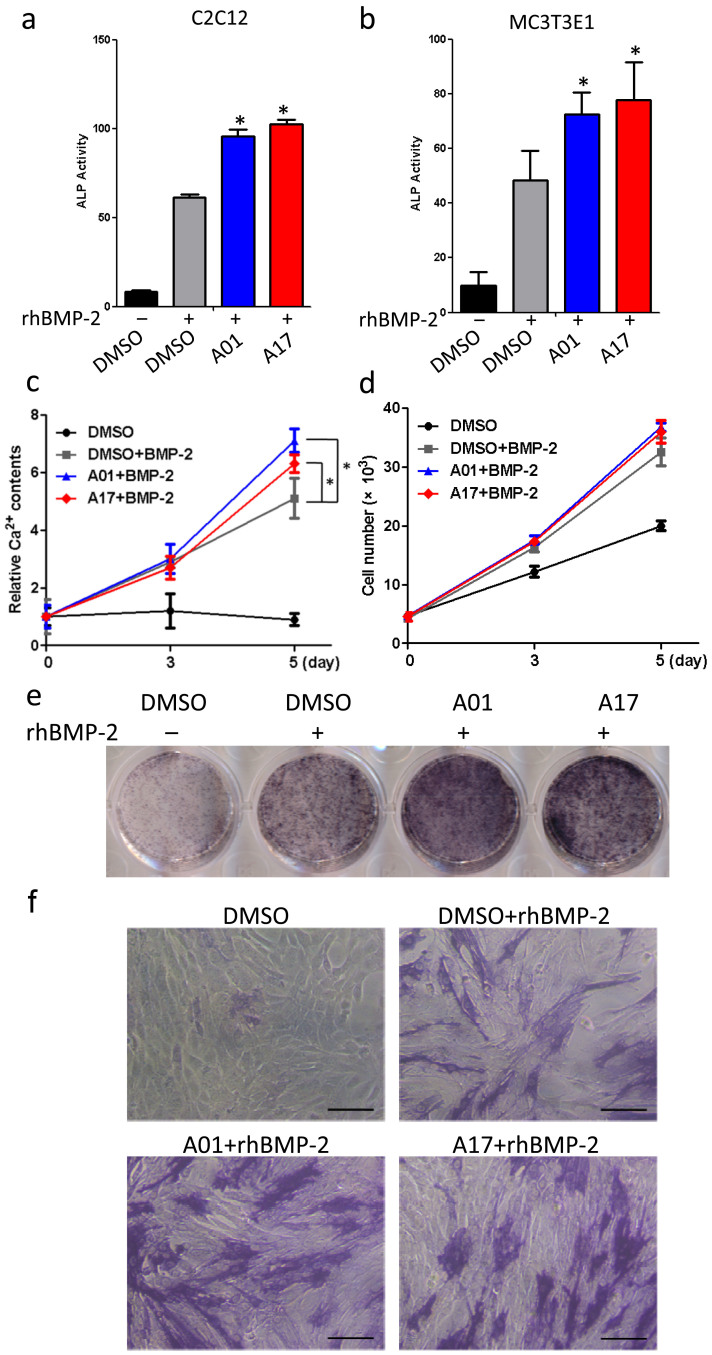
Selective compounds potentiate BMP-2 induced osteoblastic activity. (a) Selective compounds increased BMP-2 induced ALP activity in mouse myoblasts. C2C12 cells were treated A01 and A17 at 2 μM, while rhBMP-2 was used at 50 ng/ml. Data points were determined in triplicate and showed with the mean ± SD (*: p < 0.05, t-test). (b) Selective compounds increased BMP-2 induced ALP activity in mouse pre-osteoblasts. MC3T3-E1 cells were treated A01 and A17 at 2 μM, while rhBMP-2 was used at 50 ng/ml. Data points were determined in triplicate and showed with the mean ± SD (*: p < 0.05, t-test). (c) Selective compounds enhanced BMP-2 induced intracellular Ca^2+^ accumulation. C2C12 cells were treated A01 and A17 at 10 μM, while rhBMP-2 was used at 100 ng/ml. Data points were determined in triplicate and showed with the mean ± SD (*: p < 0.05, t-test). (d) Selective compounds mildly facilitated BMP-2 induced cell proliferation. C2C12 cells were treated A01 and A17 at 10 μM, while rhBMP-2 was used at 100 ng/ml. Data points were determined in triplicate and showed with the mean ± SD. (e) Selective compounds potentiated BMP-2 induced ALP enrichment. C2C12 cells were treated A01 and A17 at 10 μM, while rhBMP-2 was used at 100 ng/ml. (f) ALP staining results under microscope. Scale bar: 100 μm.

The validity of selective compounds was confirmed in both C2C12 and MC3T3-E1 cells by testing the ALP activity after compounds administration. In C2C12 cells, at 2 μM, A01 and A17 elevated ALP activity by almost one fold after 48 hours (Figure 6a). In MC3T3-E1 cells, both selected compounds had the similar effects (Figure 6b). Furthermore, due to the future need of drug evaluation in ovariectomized rat osteoporotic model, the effect of selective compounds on ALP activity was tested in rat osteoblasts-like cells ROS17/2.8 and a significant effect of both candidates were observed (Supplementary Figure S8). Administration of selective compounds by a concentration ladder drew a gradually increased ALP activity in C2C12 cells, indicated that this elevation of ALP activity was specifically induced by A01 and A17 (Supplementary Figure S9). Then BMP-2 induced osteoblastic differentiation of C2C12 cell was scheduled to further determine the effects of A01 and A17 on osteoblastic activity. After a 5 days administration, both compounds potentiated the intracellular Ca^2+^ accumulation significantly comparing with the control group (Figure 6c); they also slightly increase cell proliferation upon administration (Figure 6d). Further, ALP staining was performed on day 5. While BMP-2 significantly elevated intracellular ALP content, A01 and A17 treatments together with BMP-2 could farther enhance it (Figure 6e). Through microscopy, the increase of ALP content can be easily observed (Figure 6f). Next we tested whether selective compounds would affect regular cell survival. Cytotoxic analysis showed that A01 reduced cell viability to 80% at 10 μM while A17 at the same concentration reduced cell viability to almost 70% (Supplementary Figure S10). Moreover, ten hours after simultaneously administrated both compounds along with rhBMP-2 to cells which were released from G1 phase, slight increase of S phase cell population could be detected (Supplementary Figure S11). Since accelerated cell proliferation is a sign of enhanced osteoblastic activity, this observation indicates a positive impact of both compounds on BMP-2 signaling.

## Discussion

By regulating the protein turnover, Smurf1 exhibits versatile functions in multiple signal pathways involved in various biological processes, such as bone formation, embryonic development, cell polarization, cell migration and adhesion, viral autophagy, and immune responses^23^. The research interest of the role Smurf1 plays in BMP pathway has driven investigations to uncover its new substrates and regulatory mechanisms. Smurf1−/− mice showed age-dependent BMD increase without other abnormality^11^. In rodent models, down-regulating the E3 activity of Smurf1 by knocking-out its activators CKIP-1 and Cdh1 enhanced osteogenetic activity^24^,^25^; while up-regulating its protein level by over-expression of itself or knocking-down its ubiquitin ligase SCF^FBXL15^ led to bone formation defects or postnatal dwarfism^26^,^27^. In osteoblasts, converting wild-type Smurf1 into a catalytic mutant (C699A), which loses the E3 catalytic ability, increased its osteoblastic ability^20^. These results suggest that Smurf1 plays a negative role in BMP signaling and osteogenesis, indicates that Smurf1 disruption could be an eligible therapeutic strategy to increase BMP signal responsiveness, and consequently, to address clinically complicated bone volume disorders such as osteoporosis and impeded fracture repair.

There are two main strategies to disrupt normal Smurf1 functions: first is to reduce the mRNA or protein level of Smurf1; and second is to specifically down-regulate its E3 activity or its E3-substrate interactions. A recent work reported that a micro-RNA named Mir-17 blocks Smurf1 expression by binding to its 3′UTR, therefore increases osteoblast differentiation^28^. Although RNA-based therapeutics may play an indispensable role in future clinical medication, currently the inherently unstable and potentially immunogenic properties set a long way for it to develop^29^. The catalytic core of Smurf1 locates at the C lobe of the HECT domain; a Cys699 residue forms the thioester bond with the ubiquitin during ubiquitination. Obviously, blocking the ubiquitin binding ability of this catalytic core would interrupt the E3 activity of Smurf1. Canonically, Smurf1 binds to substrates by the WW domains, and in some cases by its C2 or HECT domain^23^. By structure modeling of Smurf1 WW2 domain and computational screening, Satoshi Kato and colleagues identified a compound SVAK-12 which might interrupt Smurf1-substrate interaction and increase BMP signaling^3^. After the structure of Smurf1-Smad1 interaction has been obtained via nuclear magnetic resonance (NMR), more detailed studies could be carried out to find a solution to specifically block Smurf1 capturing activated R-Smads. Here, we analyzed the co-structure of Smurf1 WW domains and Smad1 interaction. Since the interaction between the WW1 domain and diphosphorylated Smad1 linker region determines the affinity of Smurf1 to activated Smad1, we identified a binding hydrophobic pocket, which provides a place for this interaction, based on which we performed further *in silico* hits screening.

Modern pharmaceutical industry and academic research to discover novel bio-active compounds have developed from high-throughput screening (HTS), fragment-based drug discovery (FBDD) to computer-aided drug design (CADD). Nowadays, because the multidimensional NMR and crystallography provide plentiful structural information of promising biological targets, structure-based CADD has gained more ground^30^. CADD efficiently avoids the high cost of maintaining a large physical compound library, economizes the time consumption in onerous experimental operation and enhances the chemical diversity of the virtual compound pool. However, regardless of which kind of algorithm or how fast one software employs or calculates, the inherent weakness of virtual screening remains that it can only fuzzily guide the following physical screening but not offer the real bio-active compounds due to the potential vital errors may generate during imitating the real chemical interaction^31^. Hence, in spite of spending much time on computing, the highly accurate computing result will surely reduce the demanded compounds amount in the second screening. We chose eHiTS to perform virtual screening because of its advantage in internal scoring system, which combines knowledge-based, statistics, the empirical approaches along with entropy loss estimation and grid based geometrical terms^32^. The major advantage of our in silico screening is: we enriched the initial compound pool by combining the product catalogs form four chemistry industrial companies, which ensured the chemical diversity that approx a million compounds still remained after fore-performed druggability selection. With the large and chemical diverse pool, we can easily obtain more potential hits that bind to the defined hydrophobic pocket without many ones that do not satisfy the druggability rules^33^. Our binding mode analysis was crucial due to that it filtered out a group of hits that have high possibility to bind to the target pocket.

To preferably fit our aim, we chose ALP activity as the elimination standard. This approach helped us to directly find the bio-active candidates for further examination. A01 and A17 stand out. Encouragingly, both selective compounds obviously interfered Smurf1 mediated Smad1/5 degradation, result in the stabilization of Smad1/5 protein and stable phosphorylation level, and increase ALP activity and the expression level of bone formation related genes. We chose mouse myoblast cell line C2C12 because it is used widely for BMP-2 induced osteoblastic differentiation^34^. However, there are many unsolved questions in our work. Firstly, endogenous Smurf1 can ubiquitinate the activated Smad1/5 (diphosphorylated by CDK and GSK), but after over-expression, exogenous Smurf1 can degrade non-activated Smad1/5. This may because that the more than ten folds up-regulation of Smurf1 protein level would blanket the affinity gap between non- and di-phosphorylated Smad1/5 with Smurf1. This speculation partly caters to the expectation that A01 and A17 can block the interaction between WW1 and Smad1/5. Secondly, after treated with the proteasome inhibitor MG132, the protein level of Smad1/5 increased without rhBMP-2 stimulation, which leads to a remaining question: what is the degradation mechanism of non-activated Smad1/5? A recent study raised the same query^35^, and make it clear should help us to perform more elaborated investigation. Thirdly, Runx2 is a well identified substrate of Smurf1, and has been proved as a master transcriptional co-factor during osteoblastic differentiation. Although it is known that Smurf1 captures RunX2 by WW-PY interacting, in this work, both selected compounds exerted notable effects on Runx2, but the effects are not pronounced as Smad1/5. The unexpected result may attribute to two reasons: one, Smurf1 mediated Runx2 degradation may be induced by other stimulus independent of BMP-2^20^,^36^; second, Runx2 has other ligases besides Smurf1, as even full abolishment of Smurf1 cannot entirely regulate Runx2 protein stability^37^,^38^.

**Figure 7:**
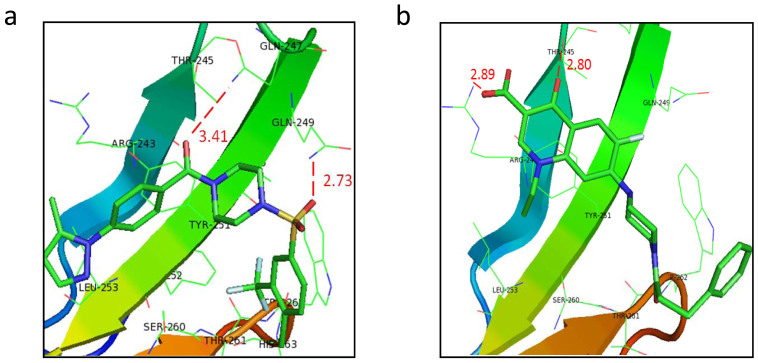
Predicted binding modes of selective compounds to the defined pocket. (a) Predicted binding modes of A01 with the defined pocket. Important residues in the pocket were labeled in black. The distances (angstrom) of hydrogen bonds donors and receptors were noted in numerical values and red dotted lines. (b) Predicted binding modes of A17 with the defined pocket. Important residues in the pocket were labeled in black. The distances (angstrom) of hydrogen bonds donors and receptors were noted in numerical values and red dotted lines.

Structure-activity relationship (SAR) study has been extensively employed to correlate molecular structures to their biological activities^39^. A better understanding of how compounds interact with the defined hydrophobic pocket will be helpful for further derivatives design and off-target effect prediction. After molecular auto-docking, the binding manners of both selective compounds are calculated and predicted, which will benefit future investigations. A01 forms two hydrogen bonds with the WW1 domain, the dominant one from the sulfonyl to the residue Gln249, the weaker one from the carbonyl to the residue Gln247 (Figure 7a). Both hydrogen bonds may be important for positioning the main piperazine ring, terminal pyrazol and benzene rings proximally to the hydrophobic pocket, thereby facilitating the holistic covalent bond formation. A17 forms two hydrogen bonds with the first β sheet of the WW1 domain, from the oxo and carboxylic acid motifs to the residues Thr245 and Arg243 respectively, which may help the main dihydroquinoline moiety embeding optimally into the hydrophobic pocket (Figure 7b). Moreover, finding out the SAR of the compounds will help researchers to conclude an agreeable degree of which properties are determinant in the mechanism of pharmacological effect^40^, thus can guide the following modification of bio-active compounds. Further, we need to study on the experimental SAR of A01 and A17, and try to find an efficient strategy for chemical modification and next generation compounds design.

Osteopenic disorders result in severe heath and social challenges that the high cost and difficulties of fracture repair, inflammatory bone loss and osteoporosis treatments are still bothering patients and the whole society. Currently, the main group of pharmacological therapy is anti-resorptive (anti-catabolic) drugs, such as estrogen, selective estrogen receptor modulators (SERM), calcitonin, biphosphonates and etc.^41^. However, these drugs do not lead to accrual of bone in the skeleton, despite the fact that bone mineral density is increased^41^. The only approved drug that stimulates bone formation, parathyroid hormone (PTH) was reported to stimulate bone resorption while increasing bone mineral density^42^. All the facts appeal novel strategies of developing bone anabolic drugs. For the important role BMP-2 plays in bone formation, rhBMP-2 was approved as a bone graft substitute to achieve fracture repair in clinical use. However, as discussed above, the gap between the high required concentration in human use (1.5 mg/ml) and the much lower one in cell culture (100 ng/ml) creates a vast barrier for routine clinical application due to the difficulty of protein preservation and high cost^4^,^6^. Thus, finding a solution to increase BMP-2 signal responsiveness that reduces basal concentration of rhBMP-2 in clinical use fits the aim of the new drug development. Moreover, since the ubiquitin-proteasome system (UPS) plays an extensive but (functionally) unique role in biological processes, E3s are vastly feasible in the specificity and sensitivity of substrate selection^43^. However, rare efforts of targeting to the UPS have been put into BMP signaling or osteopenia related disorders. Thus, drugs designed to enhance BMP responsiveness by selectively blocking UPS mediated degradation of effective pathway components may not only provide an avenue of achieving induced bone formation but also hew out a new pharmaceutical strategy, through targeting the UPS, for treating osteopenic disorders that may be further combined efficiently with other approaches of different pharmacological effects. Our results showed that selective compounds A01 and A17 can inhibit Smurf1-mediated Smad1/5 degradation and accelerate BMP-2 signal responsiveness at low dose, indicating that pharmacologically targeting Smurf1 for inhibition is promising for increasing BMP signaling activity. Further, our experience might probably provide new thoughts of selecting small molecule compounds that specifically antagonize certain ubiquitin ligase's activity for increasing the cellular signal transduction efficiency.

## Methods

### *In silico* screening

The structures of WW1-Smad1 interactions were obtained from Protein Data Bank, PDB IDs are: 2LAZ (mono-phosphorylated Smad1) and 2LB0 (diphosphorylated Smad1). Next, to guarantee chemically diverse a large pool comprising greater than two million compounds was integrated with catalogs form four chemistry industrial companies: InterBioScreen Ltd., ChemBridge Corporation, ENAMINE Ltd. and Life Chemicals Inc. Then, all compounds were evaluated the druggability by ADMET Predictor (Simulations Plus Inc. USA), on which a cutoff line was set to eliminate the compounds with ADMET risk score of ≤2, to shrink this pool to smaller virtual library of about one million compounds. The target pocket was defined by the docking program eHiTS (SimBioSys Inc. Canada), and the virtual ligand auto-docking was performed by three hierarchical steps: (1). Fast docking, accuracy parameter was 1, 1280 rigid fragments were collected for each docking. This step abated the pool to 100,000 compounds. (2). Accurate docking, accuracy parameter was 3, 6720 rigid fragments were collected for each docking. This step abated the pool to 10,000 compounds. (3). High accuracy docking, accuracy parameter was 6 (the highest one), 22080 rigid fragments were collected for each docking. This step abated the pool to 2,000 compounds. The eHiTS score (log Kd) of each ligand was generated simultaneously after these procedures. The docking conformation of each ligand and the pocket was visualized by CheVi (SimBioSys Inc. Canada). The chemical skeleton clustering was performed using MedChem Studio (Simulations Plus Inc. USA).

### Western blot, co-immunoprecipitation GST-pull down and *in vivo* ubiquitination assays

The experimental procedures of western Blot (WB), GST-pull down (GPD) and co-immunoprecipitation (Co-IP) were performed as described before^44^,^45^. For the *in vivo* ubiquitination (IVU) assays, cells were treated with MG132 (20 μM) together with the selective compounds or vehicle to avoid the proteasome-mediated protein degradation. The cell lysate was prepared in HEPES lysis buffer supplemented with protease inhibitors, and proteins were immunoprecipitated with the indicated antibody and detected by immunoblotting with anti-ubiquitin antibody. Before those assays, cells were cultured with the administration of compounds and rhBMP-2. For direct WB, cells were harvested 8 hours after drug administration; for IVU and Co-IP, cells were harvested 6 hours after drug administration (cell tranfection was performed 40 hours before chemical administration); for GPD, compounds were added with his-attached protein at various concentrations into the system, and incubated with GST-Smurf1 for 8 h at 4°C under rotation.

### Real-time RT-PCR and luciferase reporter assay

For real-time PCR, cells were administrated with compounds and rhBMP-2 for 24 hours before harvest. Total RNA was isolated from the cells using TRIzol (Invitrogen) and reversed-transcribed using 1 μg of total RNA using First-Strand cDNA Synthesis SuperMix (TransCen Biotech). The following real-time PCR was performed with the IQ5 System (Bio-Rad). PCR reactions were performed in 25 μl reactions with SYBR Green PCR master mix (Bio-Rad) and 0.2 μM specific primers, which have been listed in the supplementary materials. Transcription reporter assay in HEK293T cells was described previously^27^. Before harvest, cells were transfected with 1 μg control vector or Flag-Smurf1 for 24 hours, then add BMP-2 and compounds for 24 hours.

### ALP assays, cell counting and Ca^2+^ accumulation examination

ALP activity was examined by Alkaline Phosphatase Assay Kit (BioAssay Systems) following the manufacturer's protocols. Before these assays, cells were administrated with compounds and rhBMP-2, on the day 2, the medium was replaced by new medium and rhBMP-2. Cells were harvested on the 3th day. ALP staining was performed by the BCIP/NBT Alkaline Phosphatase Color Development Kit (Beyotime) following the manufacturer's protocols. Before these assays, the medium was replaced by differentiation medium and cells were administrated with compounds and rhBMP-2, on the day 3, the medium was replaced by new differentiation medium with rhBMP-2 and compounds. Cells were stained on the 6th day. For cell counting, cells were plated at a density of 3 × 10^3^ cells/cm^2^ and cultured in differentiation medium, then treated with compounds and rhBMP-2. At each time point, cells were counted using a haemocytometer. For Ca2+ accumulation examination, For Ca2+ content examination, cells were cultured in differentiation medium and then treated with compounds and rhBMP-2. At each time point, tests were performed by C-TEST kit (Wako) following the manufacturer's protocols.

### Molecular visualization and statistical analysis

Protein structures and docking results were visualized and plotted by PyMOL (DeLano) and statistical evaluation was conducted with student's t-test by SPSS statistics 17.0.

## Author Contributions

Y.C. and L.Z. designed the project; Y.C. and L.Z. wrote the article. Y.C. performed the experiments and analyzed the data; C.W., X.Z. and G.X. helped to perform cell culture based experiments and prepare the reagents and materials; K.L., Y.G. and F.H. contributed to the data analysis.

## Supplementary Material

Supplementary Information Supplementary information
