# Supplementary material for: Selective Small Molecule Compounds Increase BMP-2 Responsiveness by Inhibiting Smurf1-mediated Smad1/5 Degradation
Source: Sci Rep. 2014 May 14;4:4965. doi: 10.1038/srep04965 (PMC4021816; doi:10.1038/srep04965)
Supplement: Supplementary Information [file srep04965-s1.docx]

**Selective Small Molecule Compounds Increase BMP-2 Responsiveness by Inhibiting Smurf1-mediated Smad1/5 degradation**

Yu Cao^1^, Cheng Wang^1, 2^, Xueli Zhang^1^, Guichun Xing^1^, Kefeng Lu^1^, Yongqing Gu^2^, Fuchu He^1^, Lingqiang Zhang^1, 3, *^

^1^ State Key Laboratory of Proteomics, Beijing Proteome Research Center, Beijing Institute of Radiation Medicine, Collaborative Innovation Center for Cancer Medicine, Beijing, China. ^2^ School of Medicine, Shihezi University, Shihezi, Xinjiang Province, China. ^3^ Institute of Cancer Stem Cell, Dalian Medical University, Dalian, Liaoning Province, China.

^*^ Correspondence: zhanglq@nic.bmi.ac.cn

**Supplementary Table S1**

| ID | Chemical Structure | Score (kcal/mol) | ID | Chemical Structure | Score (kcal/mol) |
| --- | --- | --- | --- | --- | --- |
| A01 | **** | -8.436 | A11 |  | -7.703 |
| A02 | **** | -8.007 | A14 |  | -7.615 |
| A03 | **** | -7.918 | A15 |  | -7.616 |
| A04 |  | -7.896 | A17 |  | -7.597 |
| A05 |  | -7.886 | A18 |  | -7.566 |
| A06 |  | -7.855 | A25 |  | -7.508 |
| A07 |  | -7.849 | A54 |  | -7.316 |
| A08 |  | -7.775 | A63 |  | -7.264 |
| A09 |  | -7.766 | A75 |  | -7.211 |
| A10 |  | -7.730 |  |  |  |

**Supplementary Table S1**. Chemical structure and eHiTS score of 19 selected compounds from the top 100 high score compounds.

**Supplementary Table S2**

| Compounds ID | A01 | A17 |
| --- | --- | --- |
| Chemical Formula | C_22_H_20_ClF_3_N_4_O_3_S | C_25_H_26_FN_3_O_4_ |
| Molecular Weight | 512.9 | 451.5 |
| ClogP | 4.644 | 2.442 |
| LogS | -6.437 | -5.337 |
| H Donors | 0.0 | 1.0 |
| H acceptors | 4.0 | 6.0 |

**Supplementary Table S2**. Basic information of selective compound A01 and A17

**Supplementary Figure S1**

**
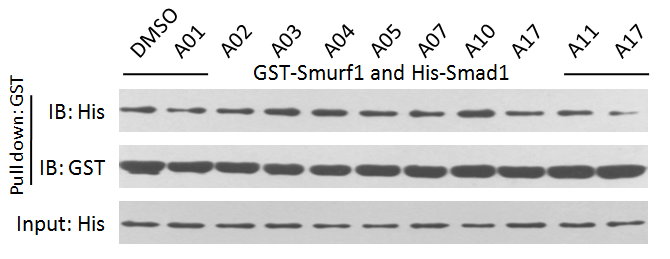

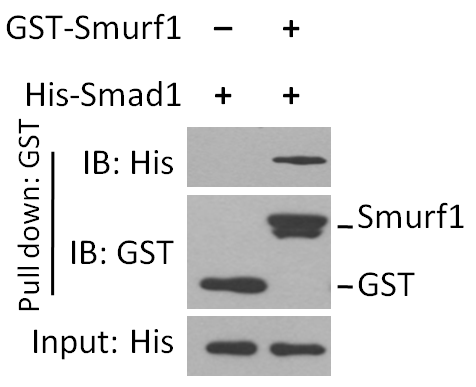
**

**Supplementary Figure S1. Impacts of selective compounds on Smurf1-Smad1 interaction.** Prokaryotic expressed proteins were purified and employed in GST-pull down. Each compound was added at 1μM. Note that cropped blots are shown here.

**Supplementary Figure S2**


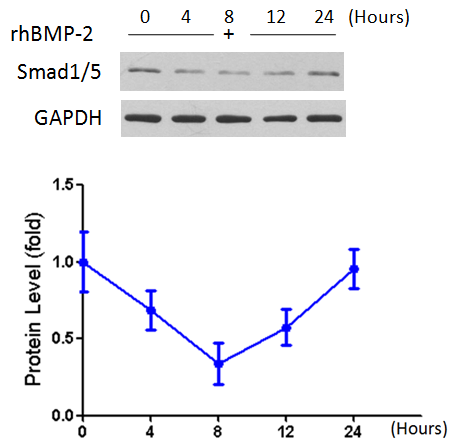


**Supplementary Figure S2. Time-dependent degradation of Smad1/5 under rhBMP-2 stimulation.** C2C12 cells were treated rhBMP-2 at 50ng/ml for various durations. Graph shows densitometry levels for percent Smad1/5 protein level and was drew by GraphPad Prism 5.

**Supplementary Figure S3**


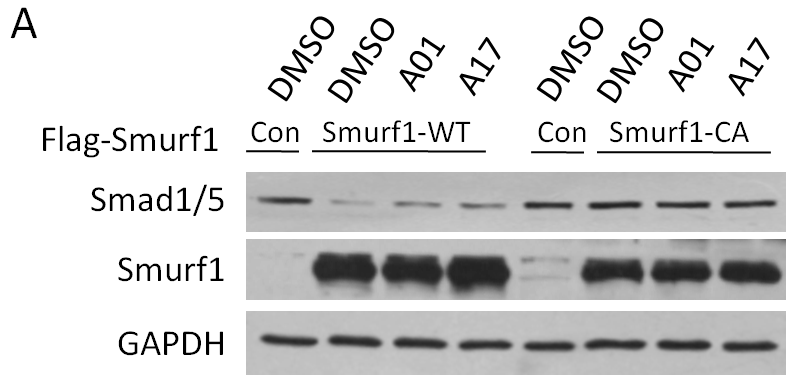


**Supplementary Figure S3. Selective compounds blocked Smurf1-medicated Smad1/5 degradation.** 293T cells were transfected Flag-empty vector (lane 1 and 5), Flag-Smurf1 (lane 2-4) and Flag-Smurf1-C699A (lane 6-8) plasmids. For inhibitors administration, cells were treated A01 and A17 at 2μM.

**Supplementary Figure S4**


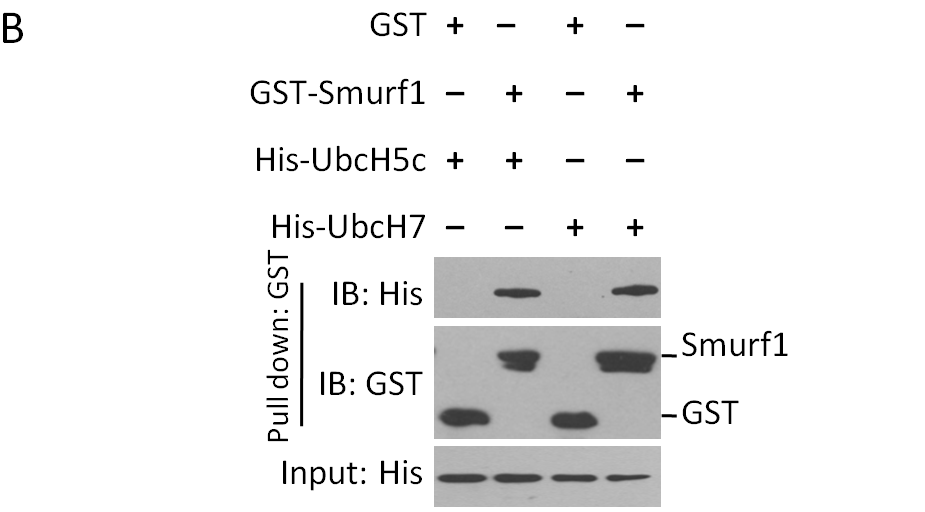


**Supplementary Figure S4. Smurf1and E2s have specific interactions.** Prokaryotic expressed proteins were purified and employed in GST-pull down. Note that cropped blots are shown here.

**Supplementary Figure S5**

**
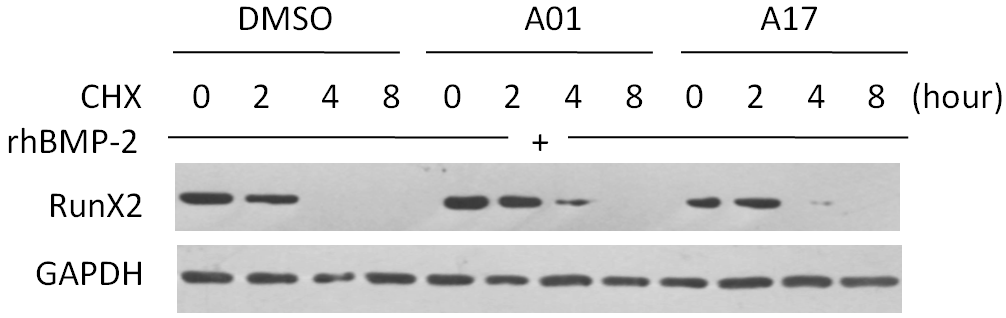
**

**Supplementary Figure S5. Selective compounds had slight effects on Runx2 protein decay.** C2C12 cells were treated with cycloheximide (CHX, 10μg/ml), rhBMP-2 (50ng/ml) and selected compounds (2μM). Cells were collected at different time points. GAPDH were used as loading controls. Note that cropped blots are shown here.

**Supplementary Figure S6**

**
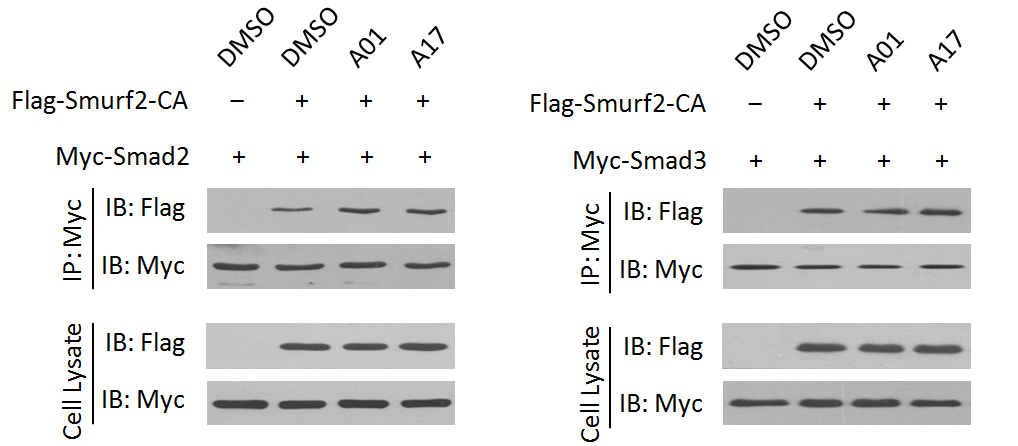
**

**Supplementary Figure S6. Selective compounds did not affect Smurf2-Smad2/3 interactions.** Left: 293T cells were co-transfected Flag-empty vector and Myc-Smad2 (lane 1) or Flag-Smurf2-CA and Myc-Smad2 (lane 2-4) plasmids. Right: 293T cells were co-transfected Flag-empty vector and Myc-Smad3 (lane 1) or Flag-Smurf2-CA and Myc-Smad3 (lane 2-4) plasmids. For inhibitors administration, cells were treated A01 and A17 at 2μM. Note that cropped blots are shown here**.**

**Supplementary Figure S7**

**
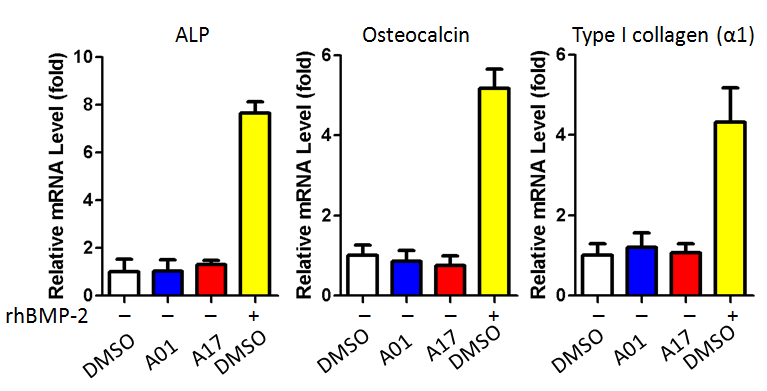
**

**Supplementary Figure S7. Selective compounds did not affect ALP, osteocalcin and type I collagen (α1) expression without BMP-2 stimulation.** C2C12 cells were treated A01 and A17 at 2μM, while rhBMP-2 was used at 50ng/ml. Data points were determined in triplicate and showed with the mean ± SD (*: p<0.05, t-test)

**Supplementary Figure S8**

**
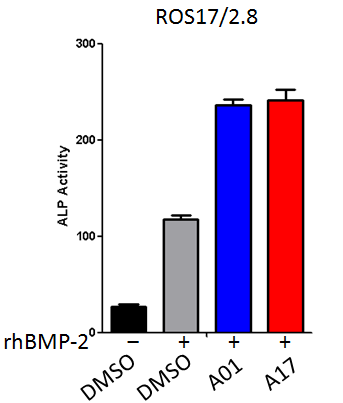
**

**Supplementary Figure S8. Selective compounds increased BMP induced ALP activity in rat osteoblasts-like cells.** ROS17/2.8 cells were treated A01 and A17 at 2μM, while rhBMP-2 was used at 50ng/ml. Data points were determined in triplicate and showed with the mean ± SD (*: p<0.05, t-test).

**Supplementary Figure S9**

**
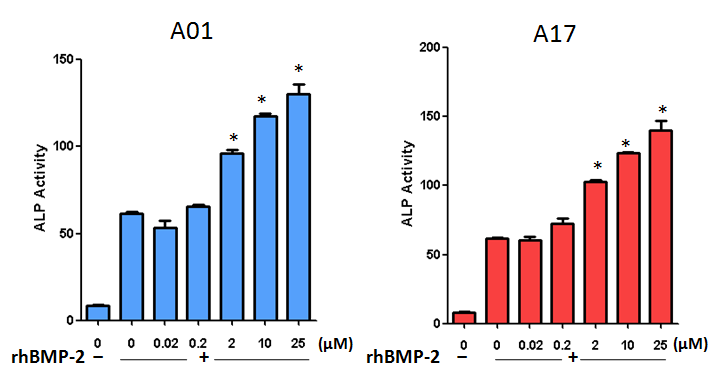
**

**Supplementary Figure S9. Selective compounds increased BMP induced ALP activity in a dose-dependent manner.** C2C12 cells were treated A01 and A17 at incremental concentrations, while rhBMP-2 was used at 50ng/ml. Data points were determined in triplicate and showed with the mean ± SD (*: p<0.05, t-test).

**Supplementary Figure S10**


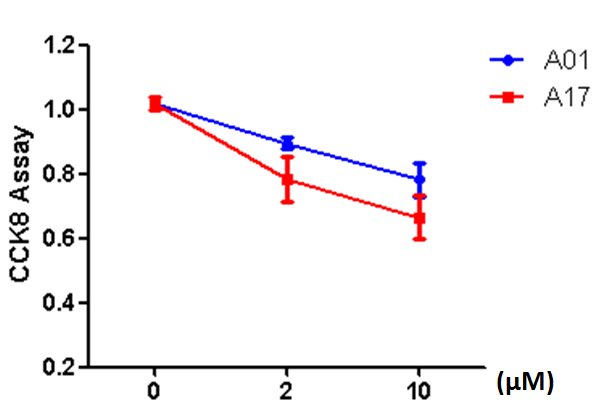


**Supplementary Figure S10. Effects of selective compounds on cell viability.** C2C12 cells were treated A01 and A17 at 2μM for 24 hours, then performed CCK8 assay.

**Supplementary Figure S11**


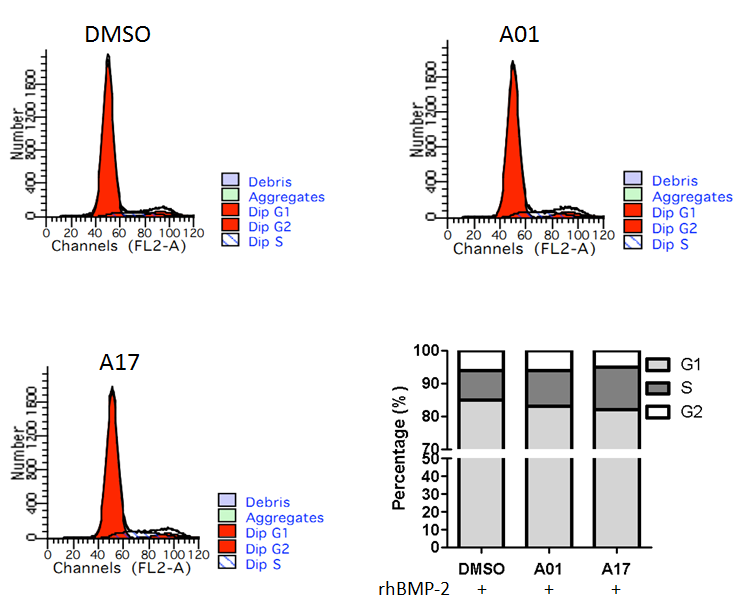


**Supplementary Figure S11. Selective compounds had slight impact on cell cycle.** C2C12 cells were were synchronized by nocodazole treatment for 24 hours, then treated A01 and A17 at 2μM while rhBMP-2 was used at 50ng/ml for 24 hours.

**Supplementary Materials**

**Antibodies and Reagents**

The anti-Myc antibody was purchased from BD Biosciences. the anti-flag and anti-ING2 antibodies were purchased from Sigma. The anti-Runx2, anti-GAPDH and the anti-MEKK2 antibodies were purchased from Santa Cruz. The anti-His and anti-GST antibodies were purchased from Tiangen Biotech. The anti-Smurf1, anti-Smad1/5, anti-RhoA, anti-Smad4, anti-Smad2/3 and anti-pSmad1 (S206) antibodies were purchased from Abcam. The anti-mouse and anti-rabbit secondary antibodies were purchased from Jackson ImmunoResearch. A01 and A17 were bought from ENAMINE Ltd (A01：T5774051；A17：Z203242870). The proteasome inhibitor MG132 and the protein synthesis inhibitor cycloheximide (CHX) were bought from Sigma. The rhBMP-2 was bought from PeproTech Inc.

**Cell Culture and Transfection**

C2C12 cell line was provided by Professor Lingling Zhu (Beijing Institute of Basic Medical Sciences), ROS17/2.8 cell line was provided by Professor Ningsheng Shao (Beijing Institute of Basic Medical Sciences), MC3T3-E1 cell line was provided by Professor Zikuan Guo (Beijing Institute of Radiation Medicine), C2C12, ROS17/2.8 and 293T cells were cultured in DMEM (Corning) supplemented with 10% fetal bovine serum (Hyclone), M3T3E1 cell was cultured in α-MEM (Corning) supplemented with 10% fetal bovine serum (Hyclone). Plasmids and siRNA were transfected into cultured cells with Lipofectamine 2000 following the manufacturer’s protocol (Invitrogen). Mouse Smurf1 specific siRNA (5’-CUCAACCGACACUGUG

AAATT-3’) and non-targeting siRNA (5’- UUCUCCGAACGUGUCACGU-3’) were synthesized by Shanghai GenePharm. The Flag-Smurf1-WT, Flag-Smurf1-CA plasmids were demonstrated previously ^1^, the GST-Smurf1, Myc-Smad1, His-Smad1, His-E2s Flag-Smurf2-CA, Myc-Smad2 and Myc-Smad3 were constructed by PCR, followed by subcloning into various vectors.

**Real-time PCR primers**

Mouse GAPDH: forward, 5’-GGGAAGGTGAAGGTCGGAGT-3’ and reverse, 5’-TTGAGGTCAATGAAGGGGTCA-3’.

Mouse Smad1: forward, 5’-TCTGAAGTGGGCTTTCATCA-3’ and reverse, 5’-TATGCCTGCCATCATTCTGA-3’.

Mouse Smad5: forward, 5’-TAGGCGGCATATTGGAAAAG-3’ and reverse, 5’-CCAGAAGCTGAGCAAACTCC-3’.

Mouse type I collagen: forward, 5’-TCGGGCCTGCTGGTGTTCGTG-3’, and reverse, 5’-TGGGCGCGGCTGTATGAGTTCTTC-3’.

Mouse ALP: forward, 5’-CACGCGATGCAACACCACTCAGG-3’, and reverse 5’-GCATGTCCCCGGGCTCAAAGA-3’.

Mouse osteocalcin: forward 5’-ACCCTGGCTGCGCTCTGTCTCT-3’, and reverse 5’-GATGCGTTTGTAGGCGGTCTTCA-3’.

**Cytotoxic and Cell Cycle Analysis**

Cell viability was measured using a tetrazolium salt (WST-8)-based colorimetric assay in the CCK-8. Briefly, cells were seeded on 96-well plates. Then treated the cells with compounds at various concentrations for 8h, spent medium was replaced with fresh medium containing 10ml CCK-8 solution, and the plate was incubated for 50 min. Cell viability was detected by scanning with a microplate reader at 450 nm. Cell Cycle analysis of C2C12 cells were synchronized by nocodazole for 24h, and then were treated with selected compounds for 8h before collecting. Cells were trypsinized, fixed in 70% ethanol in ‒20℃ for 24 h, washed and stained with propidium iodide (0.05 mg/ml; Sigma) and RNase A (0.1 mg/ml; Sigma) for 30 min at 37℃. Samples were then analyzed for their DNA content by FACSCalibur from BD Biosciences.

**Supplementary References**

1. Cui, Y.*, et al.* SCF^FBXL15^ regulates BMP signalling by directing the degradation of HECT-type ubiquitin ligase Smurf1. *EMBO J* **30**, 2675-2689 (2011).
